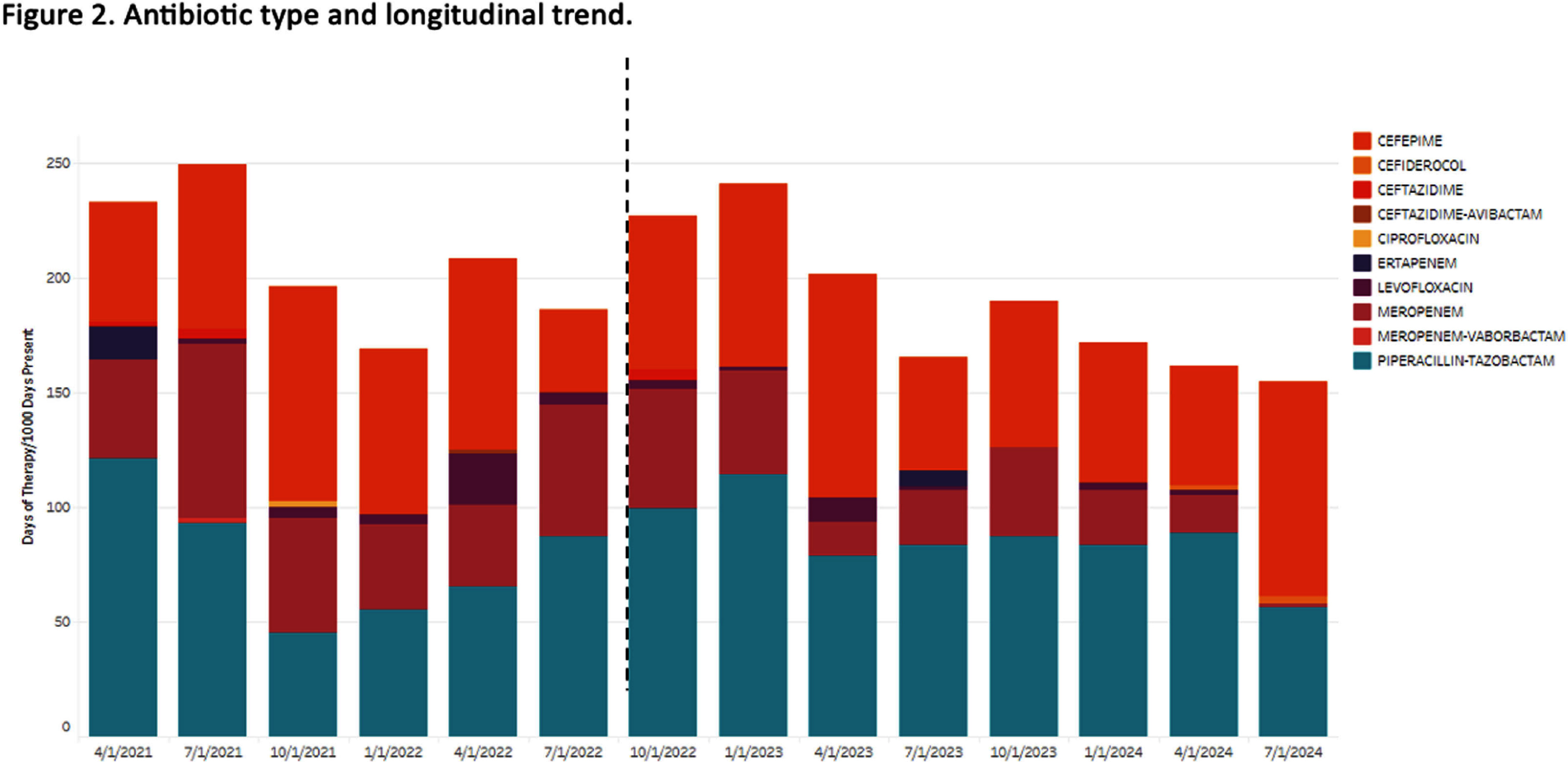# Implementation of Updated Febrile Neutropenia Guidelines Decreased Antibiotic Utilization in a Comprehensive Cancer Center

**DOI:** 10.1017/ash.2025.196

**Published:** 2025-09-24

**Authors:** Shatha AlShanqeeti, Betsy Joseph, David Riedel, Poonam Mathur, John Baddley, Sara Lee, Jacqueline Bork

**Affiliations:** 1 University of Maryland; 2 University of MD

## Abstract

**Background:** Empiric antibiotic therapy choices and de-escalation practices for the management of febrile neutropenia (FN) can vary. Facility-specific antimicrobial guidelines have an important role in influencing prescription practices for FN and is a foundation of antimicrobial stewardship activities. **Methods:** This pre-post quality improvement study at the University of Maryland Medical Center (UMMC) Greenebaum Comprehensive Cancer Center evaluated the impact of the implementation of updated institutional FN guidelines. The changes primarily included: 1) removal of meropenem as first-line agent for patients receiving levofloxacin prophylaxis without other risk factors (e.g. history of resistant organism) and 2) de-escalation protocol for low-risk patients (e.g. afebrile, hemodynamically stable). Education of oncology attendings, residents and pharmacists were carried out. We included patients receiving antipseudomonal antibiotics for FN or sepsis as indicated by prescriber (~70% concordance with antimicrobial stewardship review). Sepsis was included because of high rates of observed misclassification for patients with FN. Stem cell transplant patients were excluded. Pre-intervention (04/2021 – 12/2022) and post-intervention (01/2023 – 09/2024) groups were compared for total anti-pseudomonal antibiotic and meropenem-specific days of therapy (DOT) per 1000 days present (DP) and count of unique antibiotic order per 1000 DP. In addition, a sample of antibiotics reviewed by the UMMC antimicrobial stewardship team was assessed for guideline compliance. Means were calculated across quarters for each period and Willcoxon rank sum was used for comparisons (p Results: A total of 3,311 antibiotics were ordered for FN (79%) or sepsis (21%) during the study period. Longitudinal trends and antibiotic type distribution are illustrated in Figures 1 and 2, respectively. DOT per 1000 DP for all antipseudomonal antibiotics was 213 in the pre-intervention group compared to 191 in the post-intervention group (p=0.06). Meropenem DOT per 1000 DP decreased from 105 in the pre-intervention group to 87 in the post-intervention group (p=0.004). Unique antibiotic order per 1000 DP of all antipseudomonal antibiotics remained constant (62 vs. 56, p=0.1), while unique antibiotic order per 1000 DP for meropenem decreased (16 vs. 8, p =0.01). Of the 317 antibiotics reviewed, 130/169 (77%) were guideline compliant in the pre-intervention group and 113/148 (76%) in the post-intervention group. **Conclusion:** Changes in FN guidelines at the UMMC cancer center led to decreased meropenem use with a nonsignificant decline in all antipseudomonal antibiotics. Additional work is needed to identify barriers to guideline adherence.

**Figure:**
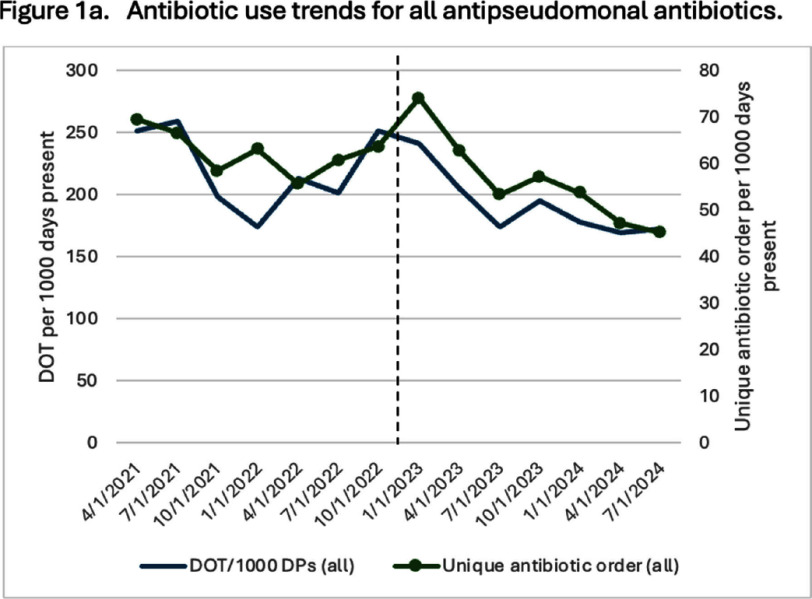

**Figure:**
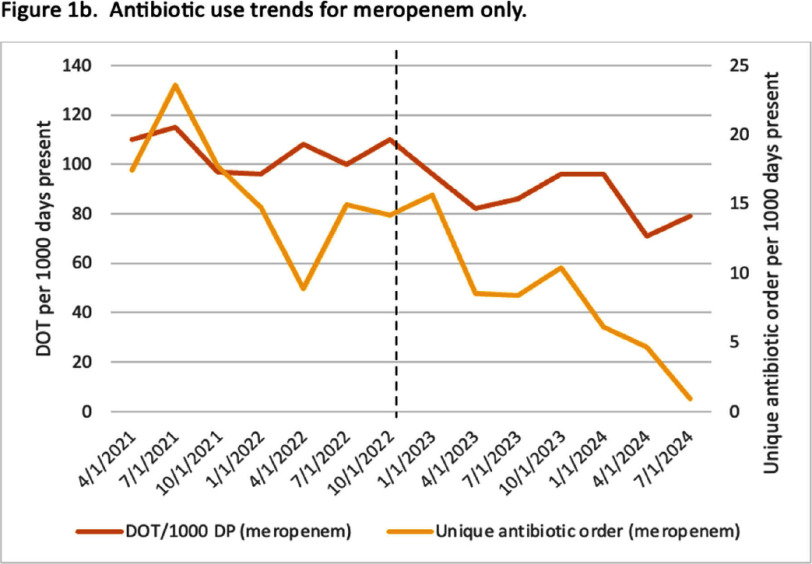

**Figure:**